# Draft Genome Sequence of *Delftia tsuruhatensis* MTQ3, a Strain of Plant Growth-Promoting Rhizobacterium with Antimicrobial Activity

**DOI:** 10.1128/genomeA.00822-15

**Published:** 2015-08-06

**Authors:** Qihui Hou, Chengqiang Wang, Haimeng Guo, Zhilin Xia, Jiangping Ye, Kai Liu, Yanan Yang, Xiaoyang Hou, Hu Liu, Jun Wang, Binghai Du, Yanqin Ding

**Affiliations:** a College of Life Sciences, Shandong Agricultural University/Shandong Key Laboratory of Agricultural Microbiology, Taian, China; b Zunyi Tobacco Company, Zunyi, China

## Abstract

*Delftia tsuruhatensis* MTQ3 is a plant growth-promoting rhizobacterium (PGPR) isolated from tobacco rhizosphere. Here, we report the draft genome sequence of *D. tsuruhatensis* MTQ3. Several functional genes related to antimicrobial activity and environment adaption have been found in the genome. This is the first genome sequence of *D. tsuruhatensis* related to PGPR.

## GENOME ANNOUNCEMENT

*Delftia tsuruhatensis*, the type species of *Delftia*, is considered a plant growth-promoting rhizobacterium (PGPR) (1–3), an organic pollutant degradation strain (4, 5), and an opportunistic pathogenic bacterium to the human body (6, 7). *D. tsuruhatensis* is widespread in rhizosphere soil, activated sludge, and polluted environments. Although some researchers have reported the antimicrobial activity of *D. tsuruhatensis*, its antimicrobial substance and relevant synthesis mechanisms are still unclear. *D. tsuruhatensis* MTQ3 was isolated from the soil of tobacco rhizosphere in Guizhou, China, and it has shown antimicrobial activity against pathogens of soilborne plant diseases such as *Ralstonia solanacearum* and *Phytophthora nicotianae*.

Here, we report the draft genome sequence of *D. tsuruhatensis* MTQ3. Genomic DNA was extracted and sequenced using the Illumina HiSeq 2500 platform. The whole shotgun sequencing produced 8,206,833 paired-end reads with an average insert size of 580 bp (over 780-fold coverage). Another mate-paired (MP) library containing 3- to 5-kb inserts was constructed, and 13,462,994 paired-end reads were produced. All these raw data were then filtered by Trimmomatic v 0.30 (8). Filtered reads were assembled, scaffolded, and gap filled with Velvet v. 1/2/08 (9), SSPACE v. 2.0 (10), and GapFiller v. 1.11 (11). Final assembly consisted of 12 scaffolds (length ≥500 bp) with an *N*_50_ length of 742,938 bp, an average length of 522,672 bp, and the largest length of 1,608,022 bp. The genome sequence was annotated using the NCBI Prokaryotic Genome Automatic Annotation Pipeline (PGAAP) (http://www.ncbi.nlm.nih.gov/genome/annotation_prok/).

The genome consists of 6.27 Mb, with a G+C content of 66.79%. A total of 5,005 coding sequences (CDS), 13 pseudogenes, 72 tRNA genes, 1 noncoding RNA (ncRNA), and 10 rRNA genes were identified. Average nucleotide identity (ANI) analysis (12) revealed that *D. tsuruhatensis* MTQ3 is phylogenetically related to *D. tsuruhatensis* 391 (98.34%) (GenBank accession no. JNWH00000000), *D. acidovorans* CCUG 274B (97.96%) (AGYX00000000), *D. acidovorans* SPH-1 (94.94%) (NC_010002), *Delftia* sp. 670 (94.30%) (JNWI00000000), *D. acidovorans* CCUG 15835 (92.70%) (AGYY00000000), and *Delftia* sp. Cs1-4 (91.37%) (NC_015563).

Many genes involved in the antimicrobial activity of *D. tsuruhatensis* MTQ3 were identified in the genome, such as the bacteriocin gene (AA671_02705), polyketide synthase (PKS) gene (AA671_12420), nonribosomal peptide synthetase (NRPS) gene (AA671_12430), balhimycin synthesis gene (AA671_12395), quinolinate synthesis gene (AA671_01765), and phenazine synthesis gene (AA671_00865). Some genes responsible for the processing and transport of antibiotics were identified in the genome, including antibiotic biosynthesis monooxygenase gene (AA671_01400, AA671_17345), macrolide transporter gene (AA671_00980), and antibiotic ABC transporter substrate-binding gene (AA671_25335).

In addition, *D. tsuruhatensis* MTQ3 possesses genes with likely relevance to rhizosphere competence and adaptation in polluted environments. Specifically, quercetin dioxygenase (AA671_16710, AA671_18600) is likely a plant microbe-signaling bacterium involved in degrading plant-produced antimicrobial root exudates (13, 14). Protocatechuate dioxygenase (AA671_06970, AA671_06975, AA671_20040, AA671_20045, AA671_25085, AA671_25090) may be related to the degradation of aromatic acid (15). Genes related to the degradation of phenylacetic acid (AA671_06085, AA671_20955, AA671_23770, AA671_24525, AA671_24535) and hydroxyatrazine (AA671_03765) were also identified.

### Nucleotide sequence accession numbers.

The whole-genome shotgun project of *D. tsuruhatensis* MTQ3 has been deposited in DDBJ/ENA/GenBank under the accession number LCZH00000000. The version described in this paper is the first version, LCZH01000000.

## References

[1] Validov S, Kamilova F, Qi S, Stephan D, Wang JJ, Makarova N, Lugtenberg B 2007 Selection of bacteria able to control *Fusarium oxysporum* f. sp. *radicis-lycopersici* in stonewool substrate. J Appl Microbiol 102:461–471. doi:10.1111/j.1365-2672.2006.03083.x. 17241352

[2] Han J, Sun L, Dong X, Cai Z, Sun X, Yang H, Wang Y, Song W 2005 Characterization of a novel plant growth-promoting bacteria strain *Delftia tsuruhatensis* HR4 both as a diazotroph and a potential biocontrol agent against various plant pathogens. Syst Appl Microbiol 28:66–76. doi:10.1016/j.syapm.2004.09.003. 15709367

[3] Jin F, Ding Y, Ding W, Reddy MS, Fernando WG, Du B 2011 Genetic diversity and phylogeny of antagonistic bacteria against *Phytophthora nicotianae* isolated from tobacco rhizosphere. Int J Mol Sci 12:3055–3071. doi:10.3390/ijms12053055. 21686169 PMC3116175

[4] Zhang LL, He D, Chen JM, Liu Y 2010 Biodegradation of 2-chloroaniline, 3-chloroaniline, and 4-chloroaniline by a novel strain *Delftia tsuruhatensis* H1. J Hazard Mater 179:875–882. doi:10.1016/j.jhazmat.2010.03.086. 20417029

[5] De Gusseme B, Vanhaecke L, Verstraete W, Boon N 2011 Degradation of acetaminophen by *Delftia tsuruhatensis* and *Pseudomonas aeruginosa* in a membrane bioreactor. Water Res 45:1829–1837. doi:10.1016/j.watres.2010.11.040. 21167545

[6] Preiswerk B, Ullrich S, Speich R, Bloemberg GV, Hombach M 2011 Human infection with *Delftia tsuruhatensis* isolated from a central venous catheter. J Med Microbiol 60:246–248. doi:10.1099/jmm.0.021238-0. 20965913

[7] Keusch S, Speich R, Treder U, Ulrich Somaini S 2013 Central venous catheter infections in outpatients with pulmonary hypertension treated with continuous iloprost. Respiration 86:402–406. doi:10.1159/000350441. 23817035

[8] Bolger AM, Lohse M, Usadel B 2014 Trimmomatic: a flexible trimmer for Illumina sequence data. Bioinformatics 30:2114–2120. doi:10.1093/bioinformatics/btu170. 24695404 PMC4103590

[9] Zerbino DR 2010 Using the Velvet *de novo* assembler for short-read sequencing technologies. Curr Protoc Bioinformatics Chapter 11:Unit 11.5. doi:10.1002/0471250953.bi1105s31. PMC2952100 20836074

[10] Boetzer M, Henkel CV, Jansen HJ, Butler D, Pirovano W 2011 Scaffolding preassembled contigs using SSPACE. Bioinformatics 27:78–79. doi:10.1093/bioinformatics/btq683. 21149342

[11] Boetzer M, Pirovano W 2012 Toward almost closed genomes with GapFiller. Genome Biol 13:R56. doi:10.1186/gb-2012-13-6-r56. 22731987 PMC3446322

[12] Richter M, Rosselló-Móra R 2009 Shifting the genomic gold standard for the prokaryotic species definition. Proc Natl Acad Sci USA 106:19126–19131. doi:10.1073/pnas.0906412106. 19855009 PMC2776425

[13] Chen XH, Koumoutsi A, Scholz R, Eisenreich A, Schneider K, Heinemeyer I, Morgenstern B, Voss B, Hess WR, Reva O, Junge H, Voigt B, Jungblut PR, Vater J, Süssmuth R, Liesegang H, Strittmatter A, Gottschalk G, Borriss R 2007 Comparative analysis of the complete genome sequence of the plant growth-promoting bacterium *Bacillus amyloliquefaciens* FZB42. Nat Biotechnol 25:1007–1014. doi:10.1038/nbt1325. 17704766

[14] Ramos FA, Takaishi Y, Shirotori M, Kawaguchi Y, Tsuchiya K, Shibata H, Higuti T, Tadokoro T, Takeuchi M 2006 Antibacterial and antioxidant activities of quercetin oxidation products from yellow onion (*Allium cepa*) skin. J Agr Foods Chem 54:3551–3557. doi:10.1021/jf060251c. 19127724

[15] Guzik U, Hupert-Kocurek K, Sitnik M, Wojcieszyńska D 2014 Protocatechuate 3,4-dioxygenase: a wide substrate specificity enzyme isolated from *Stenotrophomonas maltophilia* KB2 as a useful tool in aromatic acid biodegradation. J Mol Microbiol Biotechnol 24:150–160. doi:10.1159/000362791. 24970342

